# The influence of a major sporting event upon emergency department attendances; A retrospective cross-national European study

**DOI:** 10.1371/journal.pone.0198665

**Published:** 2018-06-13

**Authors:** Helen E. Hughes, Felipe J. Colón-González, Anne Fouillet, Alex J. Elliot, Céline Caserio-Schonemann, Thomas C. Hughes, Naomh Gallagher, Roger A. Morbey, Gillian E. Smith, Daniel Rh. Thomas, Iain R. Lake

**Affiliations:** 1 Real-time Syndromic Surveillance Team, Public Health England, Birmingham, United Kingdom; 2 Farr Institute at HeRC, Liverpool University, Liverpool, United Kingdom; 3 NIHR HPRU in Gastrointestinal Infections, Liverpool, United Kingdom; 4 NIHR HPRU in Emergency Preparedness and Response, London, United Kingdom; 5 School of Environmental Sciences, University of East Anglia, Norwich, United Kingdom; 6 Syndromic Surveillance Unit, Santé publique France, the national public health agency, Saint-Maurice, France; 7 Emergency Department, John Radcliffe Hospital, Oxford, United Kingdom; 8 Health Protection, Public Health Agency, Northern Ireland, Belfast, United Kingdom; 9 Communicable Disease Surveillance Centre, Public Health Wales, Cardiff, United Kingdom; New York City Department of Health and Mental Hygiene, UNITED STATES

## Abstract

Major sporting events may influence attendance levels at hospital emergency departments (ED). Previous research has focussed on the impact of single games, or wins/losses for specific teams/countries, limiting wider generalisations. Here we explore the impact of the Euro 2016 football championships on ED attendances across four participating nations (England, France, Northern Ireland, Wales), using a single methodology. Match days were found to have no significant impact upon daily ED attendances levels. Focussing upon hourly attendances, ED attendances across all countries in the four hour pre-match period were statistically significantly lower than would be expected (OR 0.97, 95% CI 0.94–0.99) and further reduced during matches (OR 0.94, 95% CI 0.91–0.97). In the 4 hour post-match period there was no significant increase in attendances (OR 1.01, 95% CI 0.99–1.04). However, these impacts were highly variable between individual matches: for example in the 4 hour period following the final, involving France, the number of ED attendances in France increased significantly (OR 1.27, 95% CI 1.13–1.42). Overall our results indicate relatively small impacts of major sporting events upon ED attendances. The heterogeneity observed makes it difficult for health providers to predict how major sporting events may affect ED attendances but supports the future development of compatible systems in different countries to support cross-border public health surveillance.

## Introduction

Major sporting events have the potential to influence the behaviour of the general public; wins are celebrated and losses commiserated, both locally at the event and for those following remotely (e.g. television). This public response to sporting events may have an effect on the numbers and types of attendances seen in emergency departments (EDs). The organisation of ED staffing and equipment (e.g. inpatient bed availability) in preparation for these events rely on planning assumptions, though few studies have examined the impact of major sporting events on ED attendances in detail.

Where daily ED attendances have been investigated in relation to sports events (both live and televised) there have been contrasting results; from no impact observed [1, 2], to increased assault related attendances either overall [3], or in the event of a home team win [4]. Previous investigation of daily ED attendances may have missed important hour-by-hour impacts, especially where events are of a short duration. Where potential intra-day impacts have been described there have been reported decreases directly before and during sporting events for ED attendances [5, 6] and ambulance callouts [7], as well as increases immediately following some events [6, 8]. The impact of sporting events on EDs has further been reported to differ by age, gender and reason for attendance, including; age and gender associations with violence related daily ED attendances [3, 4]; increased daily cardiovascular [9] and hourly alcohol-related [8] attendances and decreased daily and hourly paediatric attendances [10].

These earlier studies, from a range of geographical settings, employed a variety of analytical techniques and investigated a mixture of outcome variables. Here we present, to our knowledge, the most comprehensive study to date examining the impacts of a major sporting event on ED attendances. Focussing on one major tournament, the 2016 UEFA European Football Championship (Euro 2016), we use a consistent methodology across four nations involved in the tournament (England, France, Northern Ireland [NI] and Wales).

Syndromic surveillance data were used (where available) as a source of ED attendance data from multiple, geographically distinct EDs, collected using standardised methodology. Differential impacts were explored by country, time of day, age, gender and type of diagnosis. Alcohol-related and myocardial ischaemia (MI) attendances, increases in which have been linked to sporting events previously [8, 9], were readily identifiable as syndromic indicators (groupings of diagnoses codes).

## Methods

### Data sources

In England and NI the ED Syndromic Surveillance System (EDSSS) is a voluntary network of sentinel EDs covering around 10% of all emergency care attendances in England and over 30% of attendances at type 1 (major) EDs in NI during Euro 2016 [11]. The OSCOUR^®^ network in France, initially created as a voluntary network of EDs, became a mandatory national ED syndromic surveillance network during 2014. During Euro 2016 86% of all EDs in France and its overseas territories reported to OSCOUR^®^ [12]. EDs which reported to EDSSS or OSCOUR^®^ throughout the Euro 2016 period and the previous 2 years were eligible for inclusion in this study. Attendance data for all EDs in Wales were obtained using a bespoke query of the ED dataset held by the National Health Service Wales Informatics Service [13].

Overall attendance data (for any condition) were obtained for 1 June–14 July 2016, encompassing the entire period of the tournament, as well as for corresponding periods in 2014 and 2015 (matched by day of the week: 3 June–16 July 2015 and 4 June–17 July 2014), subdivided by gender and age (0–4, 5–14, 15–44, 45–64, 65+ years). The data from all EDs, were sub-divided by hour of arrival.

Using the syndromic surveillance indicators available in EDSSS (restricted to EDs recording ICD-10 [14]/ SNOMED CT [15] diagnosis codes) and OSCOUR^®^ (all EDs report ICD-10 diagnosis codes), daily data on ED attendances with a diagnosis of MI or related to alcohol (EDSSS–acute alcohol intoxication; OSCOUR^®^ –any alcohol related diagnosis) were obtained for each country, 1 June 2014–31 July 2016, plus corresponding periods in 2014 and 2015 (matched by day of the week). It is important to note that different codes were used in different countries (and indeed in different EDs), however this is most likely due to the diagnoses available for selection in the patient record, rather than a true difference between presentations. The codes available in each ED would have been likely to identify similar diagnoses (S1 Table). Wales was excluded from the syndromic indicator analysis as attendances could not be grouped by diagnosis code.

### Ethics approval and consent to participate

Ethical approval and consent to participate for this work was not required.

The anonymised EDSSS data used in this study were routinely collected and analysed as part of the public health function of PHE.

The collection and analysis of data provided by the OSCOUR^®^ network in the frame of public health surveillance and epidemiological studies has been authorized by the French National Commission for Data protection and Liberties (CNIL).

The anonymised ED data used in this study were routinely collected by NHS Wales Informatics Service and analysed as part of the public health function of Public Health Wales.

### Statistical analyses

ED attendances are influenced by hour of day, day of the week, holiday periods and time of year [16], requiring statistical analysis to differentiate between changes in attendance levels related to Euro 2016 and other effects.

Expected hourly numbers of attendances were estimated using negative binomial models, to account for possible over-dispersion in the syndromic surveillance data. The impact of matches was modelled with the inclusion of three Boolean variables (1/0) representing the ‘pre-match’ (4 hours before), ‘during match’ (2 hours: 3 hours for the final), and ‘post-match’ (4 hours after) periods. The pre-match and post-match periods were chosen based upon previous research [7, 8, 17].

Models were developed to investigate their effects on the expected hourly number of attendances, for all data pooled and for each nation individually and:

1. subdivided by weekend/ weekday;
2. subdivided by each individual game separately;
3. stratified by gender and age.

Models were specified as:

$$g(\mu_{t})=\alpha +\sum_{q=1}^{Q}\beta (x_{t})+HoD+DoW+MoY+Y+\gamma (BHol)$$

Where: g(*μ*_*t*_) is a logarithmic link function of the expectation *E(Y*_*t*_ ≡ *μ*_*t*_) (expected number of cases at time t); α denotes the intercept; *x*_*t*_ are Boolean variables indicating ‘pre-match’, ‘match’ and ‘post-match’ periods. The variables *x*_*t*_ enter the model linearly with related coefficients *β*. Potential effects of long-term and seasonal trends were controlled by categorical variables for hour of the day (*HoD*), day of the week (*DoW*), month of the year (*MoY*), and year (Y). Bank holidays (*BHol*) were accounted for with a Boolean variable (1/0) with coefficient γ.

The analysis of daily attendances for MI and alcohol-related attendances utilized a negative binomial model with the same specification. The impact of matches was modelled using Boolean variables (1/0) to represent ‘day before’, ‘match day’ and ‘day after’.

All analyses were carried out using the MASS package in R software.[18]

## Results

Euro 2016 was held in France from 10 June to 10 July 2016 [19]. Of the 51 matches played, 19 involved the England (n = 4), France (n = 7), NI (n = 4) and Wales (n = 6) national teams (Wales played matches against both England and NI).

During the study period over 2 million ED attendances were identified for the analysis of total attendances by day/hour, with the largest number of attendances from France, followed by England and Wales and much lower numbers from NI (Table 1). Attendance levels were similar between males and females (approx. 50/50) and age distributions were similar in each country, although NI included a lower percentage of paediatric attendance levels.

**Table 1:** Emergency department attendances for all causes, 1 June–14 July 2016, by nation, age and gender.

	England		France		Northern Ireland		Wales	
	(9.8% coverage)		(86% coverage)		(31.8% coverage*)		(100% coverage)	
**Total**	**286,166**		**1,837,733**		**23,966**		**127,911**	
*Males*	*143*,*005*	*50*.*0%*	*956*,*300*	*52*.*0%*	*11*,*980*	*50*.*0%*	64,320	*50*.*3%*
*Females*	*143*,*088*	*50*.*0%*	*880*,*989*	*47*.*9%*	*11*,*980*	*50*.*0%*	63,587	*49*.*7%*
*Unknown*	*73*	*0*.*0%*	*444*	*0*.*0%*	*6*	*0*.*0%*	4	*0*.*0%*
Age 0–4	26,783	9.4%	205,743	*11*.*2%*	846	*3*.*5%*	9,422	*7*.*4%*
Age 5–14	30,814	10.8%	227,616	*12*.*4%*	1,417	*5*.*9%*	16,639	*13*.*0%*
Age 15–44	113,293	39.6%	690,259	*37*.*6%*	10,675	*44*.*5%*	47,542	*37*.*2%*
Age 45–64	55,735	19.5%	345,233	*18*.*8%*	5,854	*24*.*4%*	25,963	*20*.*3%*
Age 65+	59,213	20.7%	368,882	*20*.*1%*	5,166	*21*.*6%*	28,337	*22*.*1%*
Unknown	328	0.1%	0	0.0%	8	0.0%	8	0.0%

*NI coverage of type 1 (major) ED attendances

Analysis by syndromic indicator was restricted to those EDs where detailed diagnostic coding was available, including all EDs reporting to syndromic surveillance in France and NI, a limited number of EDs in England (~6% of all ED attendances) and no data available from Wales (Table 2). France had the highest completion of diagnostic coding (91% of attendances coded). MI attendances compromised 1% of attendances in England and NI and 0.4% in France, with alcohol attendances (all alcohol-related attendances in France and acute alcohol intoxication in England and NI) accounting for 0.6%, 0.8% and 1.2% of attendances, respectively (Table 2).

**Table 2:** Emergency department attendances by syndromic indicator, 1 June–14 July 2016, by nation.

	England		France		Northern Ireland	
	(5.8% coverage)		(86% coverage)		(31.8% coverage)	
**Total Attendances**	**169,870**		**1,837,733**		**23,958**	
Diagnosis included	128,005	75.4%	1,672,825	91.0%	20,727	86.5%
*MI attendances*	*1*,*328*	*1*.*0%*	*6*,*405*	*0*.*4%*	*180*	*0*.*9%*
Alcohol* *attendances*	*758*	*0*.*6%*	*19*,*718*	*1*.*2%*	*172*	*0*.*8%*
**Mean daily attendances**						
Total Attendances	3,861		41,767		545	
*MI attendances*	*30*		*146*		*4*	
Alcohol* *attendances*	*17*		*448*		*4*	
**Estimated national mean daily attendances (calculated from coverage)**						
Total Attendances	66,563		48,566		1,712	
*MI attendances*	*520*		*169*		*13*	
Alcohol* *attendances*	*297*		*521*		*12*	

* alcohol attendances are included as ‘acute alcohol intoxication’ in the EDSSS and ‘all alcohol attendances’ in OSCOUR^®^

Similar temporal patterns in ED attendances were found in all countries. The lowest hourly attendances occurred during the night (S1 Fig) and the highest daily numbers were recorded on a Monday (S2 Fig). For the two syndromic indicators of interest (England, NI and France only) the highest alcohol-related attendances were recorded during weekends (S3 Fig) while MI attendances were highest during the week (S4 Fig).

**Fig 1:**
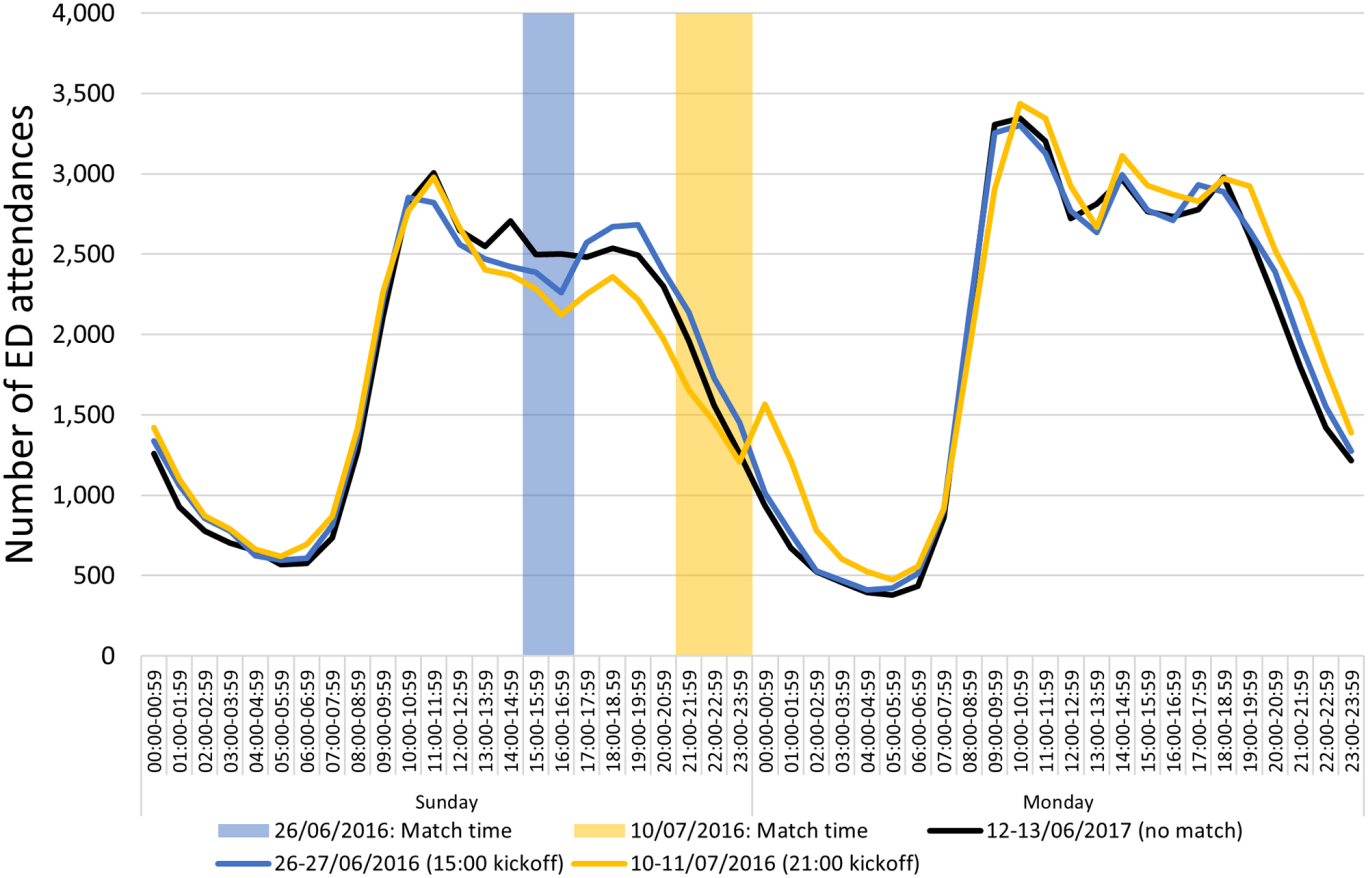
Hourly ED attendances, France, selected Sunday/Monday 48 hour periods during Euro 2016; football match times involving France are highlighted.

Descriptive analysis of temporal trends by day showed very little difference between daily numbers of attendances on match days compared with non-match days, in total and for alcohol-related or MI attendances (data not shown). Analysis of hourly attendances did, however, show some indication of reduced ED attendances during, and possible increases immediately following matches, particularly in France. The France national team played four Sunday matches during the tournament. The hourly attendances at EDs in France through Sunday afternoon into the early hours of Monday morning showed decreased attendance levels during matches and increased attendance levels immediately following matches, particularly following the afternoon match 26 July and the evening final 11 July (Fig 1). The pattern of attendances on each Monday following a Sunday match was consistent with the usual pattern observed, though possibly running an hour later on 11 July, the day after the final (Fig 1).

**Fig 2:**
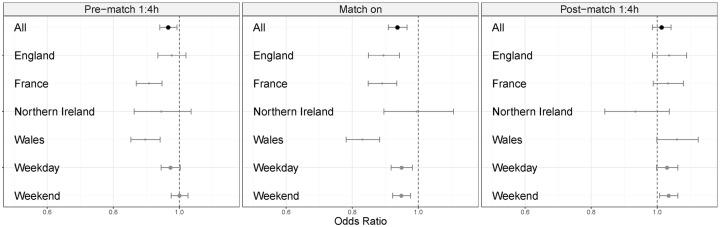
Odds ratios of ED attendance levels compared to the same time of day with no match for all matches, in total, by country and by weekday/ weekend (in total).

Overall odds ratios (OR) for hourly ED attendances (all countries) compared to the number expected indicate a statistically significant impact, although small, with pre-match and during-match reductions in ED attendances (Fig 2). Across the whole dataset (all nations, all matches) there was a reduction in pre-match attendances (OR 0.97, CI 0.94–0.99), with a stronger reduction observed on weekdays than weekends (Fig 2). Pre-match reductions were observed in each country individually and were statistically significant in France and Wales. During match periods statistically significant reductions in ED attendances were observed in all instances, though not statistically significant for Northern Ireland (Fig 2).

In the post-match 4-hour time period there was no change in ED attendances across all four countries as a whole (OR 1.01, CI, 0.99–1.04). This obscured divergence between the impacts observed in individual countries, with England, France and Wales having non-significant increases while Northern Ireland experienced reductions in ED attendances. Few differences were apparent between games played on weekends versus those played on weekdays.

**Fig 3:**
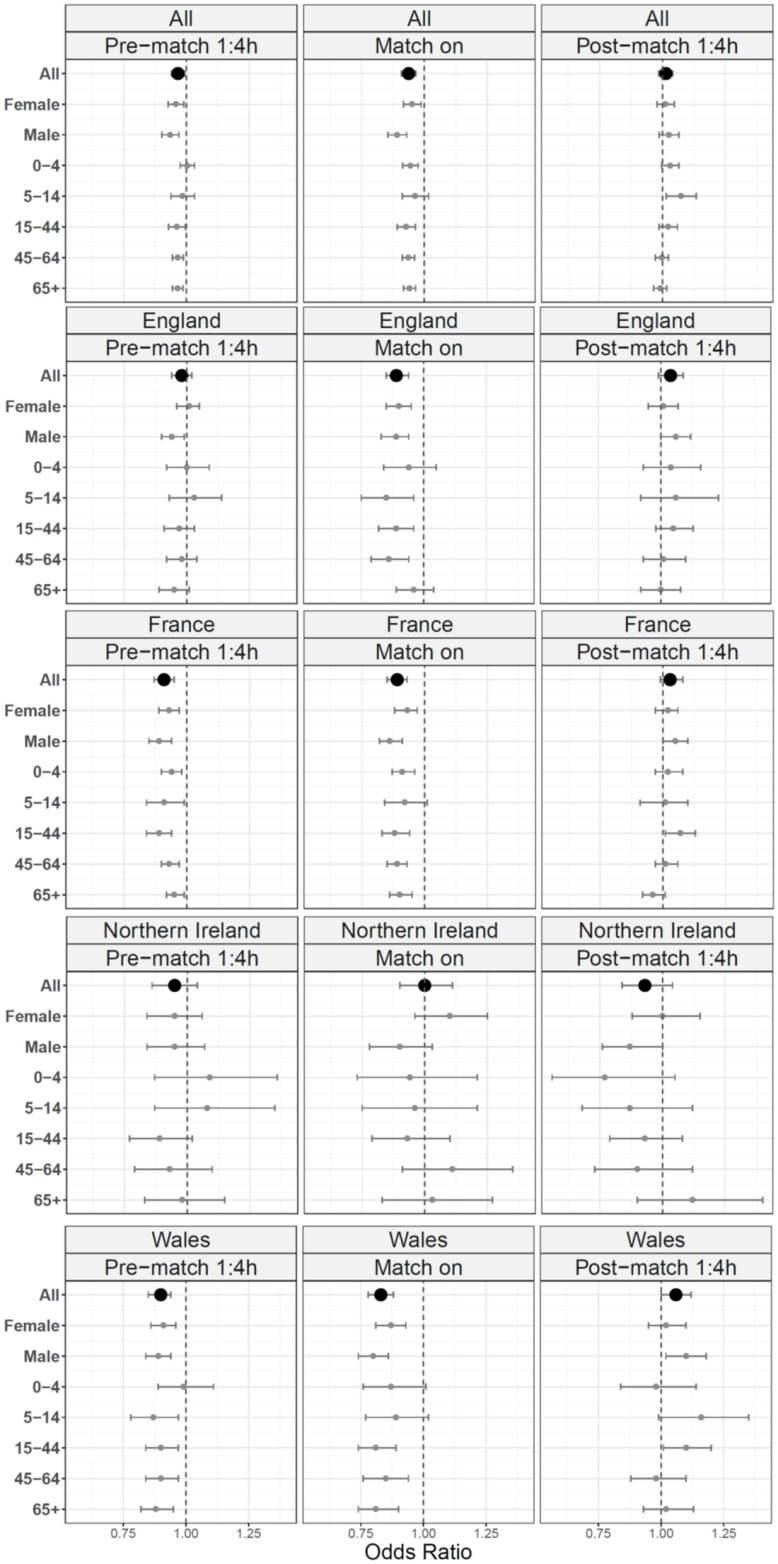
Odds ratios of ED attendance levels compared to the same time of day with no match by gender and by age group, by country.

Country specific odds ratios calculated for pre-match, during and post-match periods indicated consistency between age groups with increases/ decreases seen in all age groups together. A stronger effect was seen in school aged children (5-14yrs) and young adults (15-44yrs), with indications of a slightly larger effect in males than females (Fig 3).

**Fig 4:**
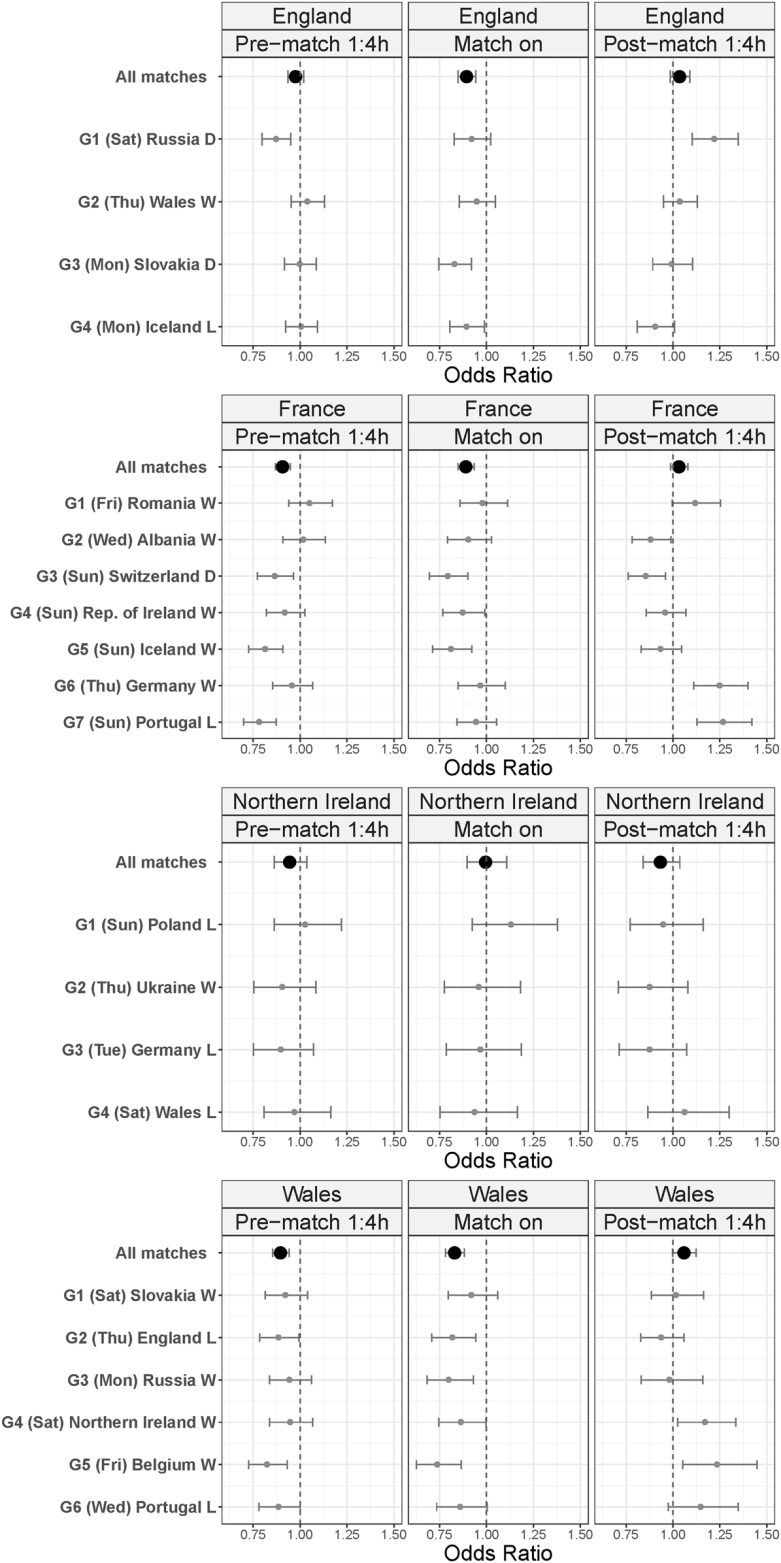
Odds ratios of ED attendance levels compared to the same time of day with no match for individual matches, by country.

Country specific ORs for individual matches showed differences between games (Fig 4). Relatively large, statistically significant drops in attendances in France were identified prior to France games 3, 5 and 7, though increases were seen prior to the first two matches. ED attendances in France during all games were lower than the same times of non-match days. The most significant post-match impacts, in terms of magnitude, were increases in attendances in France following the French semi-final (G6: OR = 1.25; CI 1.11–1.40) and final (G7: OR = 1.27; CI 1.13–1.42).

Across EDs in England, decreased attendances were observed during all matches. The biggest impact was around game 1, the only weekend game played by England (Fig 3): a relatively large decrease in attendances before the match (OR = 0.87; CI 0.8–0.95) was followed by a large increase afterwards (OR = 1.22; CI 1.1–1.35).

In Wales the greatest impact on ED attendances was during the later stages of the tournament. The first statistically significant increase in post-match attendances in Wales followed match 4, the last game of the group stage. The largest, statistically significant, pre-match/ during match reductions and post-match increases were around match 5, a quarter-final (pre OR = 0.82; CI 0.73–0.93: during OR = 0.74; CI 0.63–0.87: post OR = 1.23; CI 1.05–1.45).

No significant changes in ED attendances occurred in NI around or during matches involving the NI team.

The estimated change in total numbers of attendances for each match, varied by country though overall decreases were observed immediately before and during matches, while increases were observed immediately following matches (Table 3).

**Table 3:** Mean per match changes in emergency department attendances, in total and by country.

	Pre-match			During match			Post-match		
	Baseline	Change (%)		Baseline	Change (%)		Baseline	Change (%)	
**All Countries**	**29,798**	**-1,577**	* **(-5)**	**12,041**	**-1,247**	* **(-10)**	**14,538**	**281**	**(2)**
England	16,480	-347	(-2)	6,913	-709	* (-10)	9,429	235	(2)
France	12,193	-1,133	* (-9)	4,658	-495	* (-11)	4,487	39	(1)
N Ireland	409	-25	(-6)	182	5	(3)	280	-9	(-3)
Wales	716	-72	* (-10)	287	-47	* (-16)	343	17	(5)

* Effect significant in the statistical model presented in Fig 2a

The mean post-match increase calculated for France (1%) was the lowest of the four countries reported here. However, the two largest post-match increases were also in France, following the semi-final (France-v-Germany) and the final (France-v-Portugal; Fig 3). For these two games the nationwide increase in the four hour post-match period attendances were 772 (semi-final: 25% increase above baseline 3130 attendances) and 772 (final: 27% increase above 2912 baseline attendances). Though relatively small numbers across the country, this large percentage increase occurred late Sunday night/ early Monday morning, a time when EDs are usually quiet.

**Fig 5:**
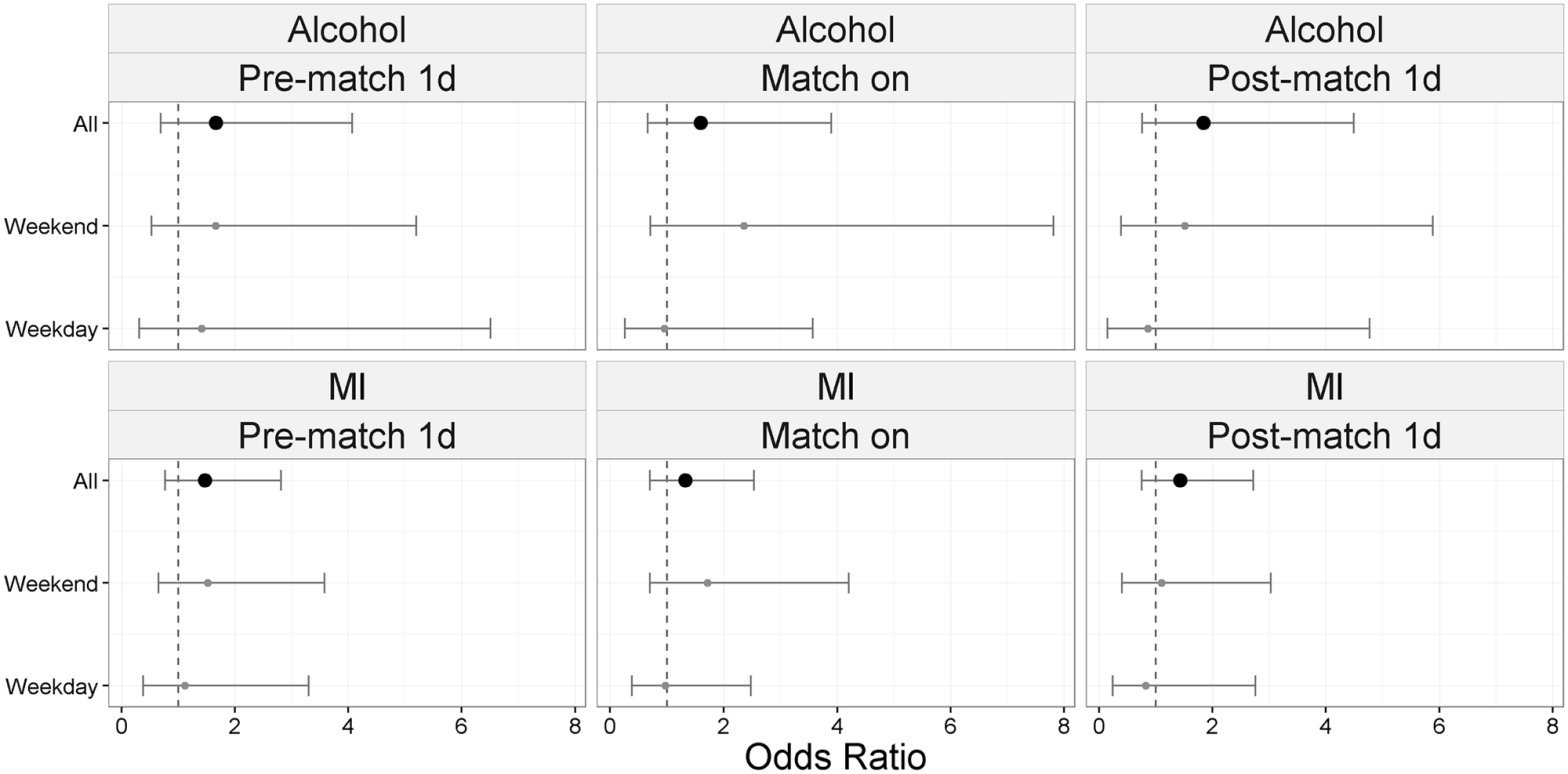
Odds ratios of daily ED attendances for alcohol and myocardial ischaemia (MI), in England, France and Northern Ireland, compared to days with no match: Pre-match, match and post-match days, by weekday/weekend.

Investigation of daily alcohol related and MI attendances showed no statistically significant changes in attendances levels, though for both indicators the ORs calculated were consistently higher at weekends than weekdays (Fig 5).

## Discussion

To our knowledge, this is the first cross-national study of the public health impact of a major sporting tournament upon ED attendances. This provides a unique insight into the impact on both the host nation, and countries with fans following the tournament from home. The use of standard public health data and a single methodology across multiple countries contrasts with previous studies which used a variety of different public health data and varying methods. This study focussed on ED attendances only, though there is no reason to believe other measures (e.g. ambulance attendances, hospital admissions) would not have shown similar results.

No significant changes in ED daily attendance levels were observed in any country, similar to the reports for England (host nation) during Euro 96 [2]. No impact was found here on alcohol-related or myocardial ischaemia daily ED attendances, however the day of the week may have influenced this result. When separated, the individual ORs for weekdays and weekends do not span the ORs for all attendances, demonstrating that weekends are a confounding factor.

Total ED attendances were further analysed by hour of attendance to identify any intra-day effects. Statistically significant decreases in ED attendances during matches were seen in all countries, except for Northern Ireland. This overall impact on EDs was estimated to be relatively minor, with minimal public health/ service impact (overall OR = 0.93; individual country ORs = 0.89–0.92, NI OR = 1). Other changes appear highly variable between countries and individual games, with wins and losses both associated in increases/ decreases in ED attendances.

The most significant impacts were demonstrated during the later stages of the tournament, particularly following the semi-final and final matches involving France. Though relatively small post-match increases in terms of actual numbers attending EDs across France (<800 during a 4-hour period), these equated to fairly large percentage increases (>25% more than would usually be expected), not necessarily evenly spread across all EDs. These particular matches ended late on a Sunday evening, with the post-match period extending into the early hours of Monday morning, a period that is usually a quiet in EDs which may be affected by even a small number of extra attendances.

The increased attendances in France following the final match of Euro 2016 contrasts with reports from a previous, similar event: during the 2003 Rugby World Cup, the host country lost in the final match, following extra time. However, following the Rugby World Cup final no significant increase in ED attendances was observed in Australia [5], contrasting with our results that ED attendances increased in France. The lack of an effect in Australia was attributed to the late end to the final match (23:00 hours) but in France the final game ended even later (00:00 hours).

The next similar football event will be the 2018 FIFA World Cup, to be staged in Russia. England and France are the only two nations included in this study to have qualified for the tournament. Looking further ahead, the Euro 2020 tournament may include all four of the nations included here, though will be hosted across 12 different European countries.[20] Each of these events do allow for the potential to carry out a follow-up study, though without the opportunity to investigate the impact on a single host country as included here.

### Strengths and limitations

The main strength of this study was the inclusion of four nations, all competing in the same sporting tournament (including the host nation), with discrete competition times and high levels of public interest. This allowed the examination of the impact of an international sporting event on a more local level.

The levels of alcohol related attendances reported here (0.6%-1.2% of attendances) are likely to have been an underestimate, since attendances where alcohol consumption was a contributing factor (e.g. injuries from falls/ fights) would not have been identified as alcohol related without an ‘alcohol’ diagnosis. The investigation of falls/ fights as a separate indicator was not possible: although the resulting diagnosis code would be readily identifiable in the ED data used here (eg wrist fracture) the mechanism by which this occurred (eg fall/fight) was not available. This type of surveillance would require much more detailed information to be available on the mechanism and intent of a presenting injury, as well as any role played by alcohol or other substances.

The pre-tournament period used for the development of model baselines was limited to 2 years due to the availability of data from the EDSSS system. This two year period was found to give the best balance between maximising the number of EDs included and the length of time available. This time period was replicated for both France and Wales to ensure a standardised methodology.

A possible limitation of this approach was that the two year pre-tournament period included the 2014 FIFA World Cup, staged in Brazil. However, Northern Ireland and Wales did not participate in the tournament, England did not perform well and did not progress past the initial group stage, with only France (of the nations included here) progressing as far as the quarter-finals of the tournament. Therefore, although the inclusion of this event may have introduced potential confounding during the baseline period developed, the failure of teams in the present study to progress to the more exciting, later matches on the 2014 World Cup implies that there will have been no discernible impact on ED attendance levels.

Another possible limitation of this study is that the possible impact of meteorological conditions on ED attendances was not controlled for. The time period included here was during the first part of the European summer, however the potential impact of heat on ED attendances was not included in the analysis. Although heat specific indicators have been developed and used to successfully identify public health impacts of extreme hot weather using ED attendances in both England and France,[21, 22] the numbers involved are generally relatively small and unlikely to affect overall attendances as investigated here, particularly when examined by hour of attendance. During the early summer period (June–mid July) in the years reported here (2014–2016) there was no evidence of extreme levels of hot weather: only a single day of ‘heatwave’ was reported in the United Kingdom (1 July 2015)[23–25] and though hotter than usual summers and several periods of ‘heat spike’ were reported in France, these were not countrywide.[26–28]

A further potential limitation is that the whole of the Euro 2016 tournament was included in the analysis for each country, introducing a possible bias caused by non-home nation games (a total of 51 games were played across the entire tournament). Matches involving other national teams may have been watched as eagerly as a home nation game (especially if the result could affect tournament progression), potentially reducing the apparent impact of home nation games. This is especially possible in France, which hosted many fans from other countries. It is also worth highlighting that initiatives specifically targeted at reducing the impact of major tournaments on the local population, such as the “Drink Less Enjoy More” campaign [29], may successfully reduce risky behaviour and the subsequent need for ED attendances.

One major challenge is understanding the human behavioural reasons behind these changes in attendances around match times. There is little research on this subject, but during the 2010 Olympic men’s ice hockey significant reductions ED attendances with lower triage severity were reported [30]. Similarly decreases in attendances with lower acuity scores at triage in the EDSSS system (England and NI) have been observed during the Christmas period [31]. Syndromic surveillance often includes the collection of indicators of severity of illness on presentation to the ED and presents opportunity for future investigation of triage severity around events/ holiday periods.

### Conclusion

In summary, we highlight that the overall influence of football games within a major European tournament upon ED attendances is relatively minor and only detectable when hourly attendances are examined. The heterogeneity between countries and the games played by individual countries (win/ lose/ draw and level of tournament progression) make it difficult for health providers to predict how major sporting events may affect ED attendances. However, the overall indication is that in the immediate build up to and during the period of an event, such as a football match, the numbers of attendances at EDs where the local population have an interest in the match (including fans watching from home) may be lower than usually expected, while attendances may increase immediately following the conclusion.

The ability to monitor the impact of large events, including sports and mass gatherings, to identify any need for public health action, is a challenge. The speed with which syndromic surveillance data is collected, processed and analysed enables changes compared to the norm to be detected in near real-time, making it a valuable tool in these situations. The implementation of compatible systems and analysis in different countries would make this possible not only locally but across international borders.

## Supporting information

S1 Table Diagnostic codes mapped to syndromic surveillance indicators included in the EDSSS (England & Northern Ireland) and OSCOUR^®^ (France) emergency department syndromic surveillance systems and used in the study. (DOCX)

S1 Fig Hourly emergency department attendances 1 June–14 July 2016, as a percentage of the total, by country. (TIF)

S2 Fig Day of the week emergency department attendances 1 June–14 July 2016, as a percentage of the total, by country. (TIF)

S3 Fig Alcohol related emergency department attendances 1 June–14 July 2016, as a percentage of attendances with a diagnosis code, by day of the week and by country. (TIF)

S4 Fig Myocardial ischaemia emergency department attendances 1 June–14 July 2016, as a percentage of attendances with a diagnosis code, by day of the week and by country. (TIF)

## Abbreviations

CI: Confidence Interval
ED: Emergency Department
EDSSS: Emergency Department Syndromic Surveillance System
Euro 2016: 2016 UEFA European Football Championship
FIFA: Fédération Internationale de Football Association
ICD-10: International Statistical Classification of Diseases and Related Health Problems-version 10
MI: Myocardial Ischaemia
NHS: National Health Service
NI: Northern Ireland
NIHR HPRU: National Institute for Health Research Health Protection Research Unit
OR: Odds Ratio
OSCOUR^®^: Organisation de la surveillance coordonnée des urgences
PHE: Public Health England
UEFA: Union of European Football Associations
