# Supplementary material for: The influence of a major sporting event upon emergency department attendances; A retrospective cross-national European study
Source: PLoS One. 2018 Jun 13;13(6):e0198665. doi: 10.1371/journal.pone.0198665 (PMC5999282; doi:10.1371/journal.pone.0198665)
Supplement: S1 Table — (DOCX) [file pone.0198665.s001.docx]

**S1 Table: Diagnostic codes mapped to syndromic surveillance indicators included in the EDSSS (England & Northern Ireland) and OSCOUR® (France) emergency department syndromic surveillance systems and used in the study**

| **Indicator** | **Country** | **Codes** | **Codesystem** |
| --- | --- | --- | --- |
| **Alcohol** | **England** | F100, T519 | **ICD-10** |
|  |  | 160573003, 160592001, 18653004, 191802004, 191806001, 222103001, 228273003, 25702006, 269765000, 2804500, 390941000000103, 42344001, 499611000000106, 53041004, 67426006, 82782008 | **Snomed CT** |
|  | **France** | F100, F1000, F1001, F1002, F1003, F1004, F1005, F1006, F1007, F102, F1020, F10200, F10201, F10202, F1021, F1022, F1023, F1024, F10240, F10241, F1025, F1026, F103, F1030, F1031, F104, F1040, F1041, Z502 | **ICD-10** |
|  | **Northern Ireland** | 25702006, 67426006 | **Snomed CT** |
| **MI** | **England** | I200, I209 ,I219, I2510 | **ICD-10** |
|  |  | 155308009, 194828000, 22298006, 233819005, 233822007, 233838001, 394659003, 398274000, 401303003, 401314000, 414545008, 414795007, 53741008, 54329005, 57054005, 59021001, 623341000000106, 67682002, 73795002, 73999000 | **Snomed CT** |
|  | **France** | I20, I200, I200+0, I201, I208, I209, I21, I210, I2100, I21000, I2108, I211, I2110, I21100, I2118, I212, I2120, I21200, I2128, I213, I2130, I21300, I2138, I214, I2140, I21400, I2148, I219, I2190, I21900, I2198, I22, I220, I2200, I22000, I2208, I221, I2210, I22100, I2218, I228, I2280, I22800, I2288, I229, I2290, I22900, I2298, I23, I230, I231, I232, I233, I234, I235, I236, I238, I24, I240, I241, I248, I249, I25, I250, I251, I252, I253, I254, I255, I256, I258, I259 | **ICD-10** |
|  | **Northern Ireland** | 155308009, 194828000, 22298006, 233819005, 394659003, 401303003, 401314000 | **Snomed CT** |
