# Supplementary figures and images for: The influence of a major sporting event upon emergency department attendances; A retrospective cross-national European study
Source: PLoS One. 2018 Jun 13;13(6):e0198665. doi: 10.1371/journal.pone.0198665 (PMC5999282; doi:10.1371/journal.pone.0198665)

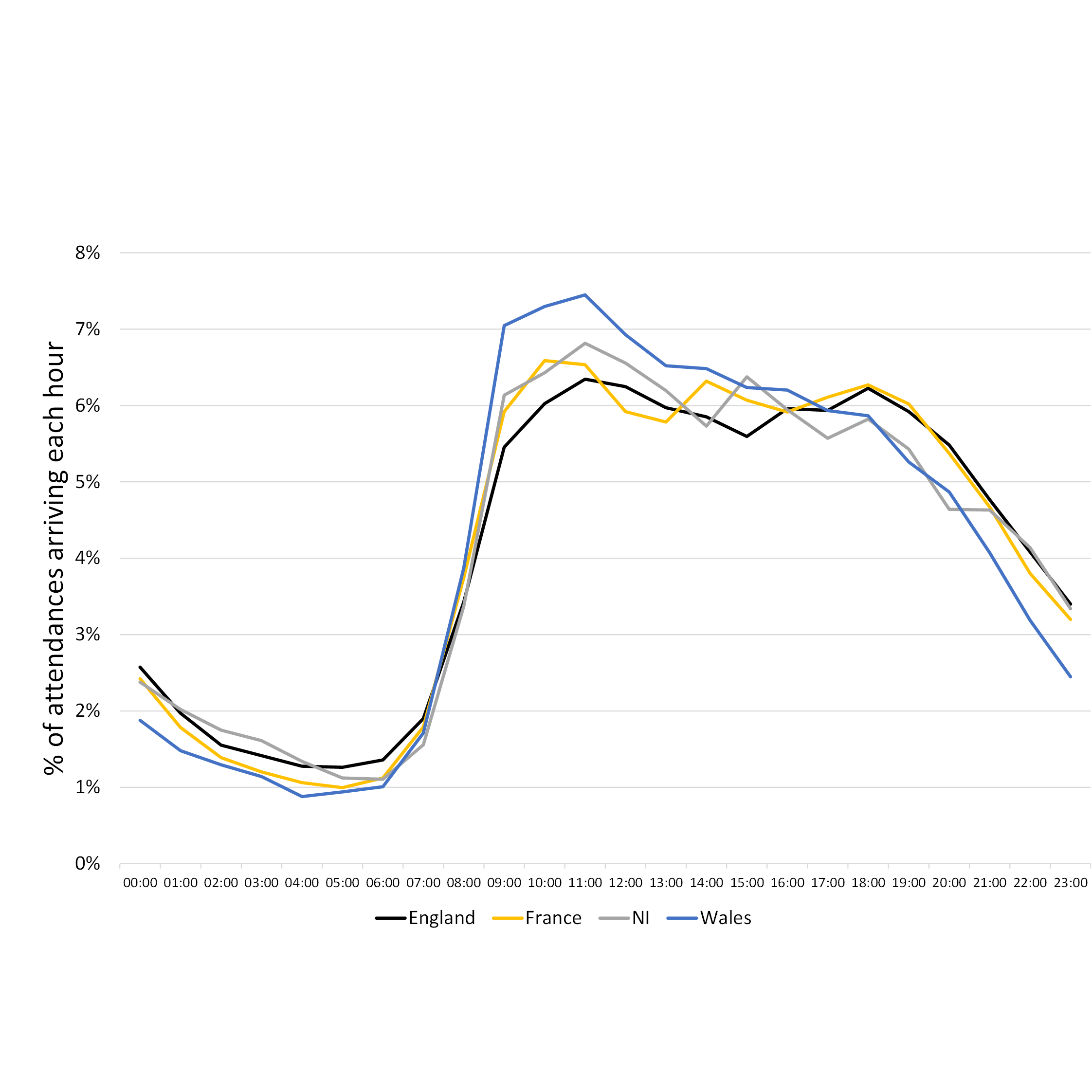

Supplement: S1 Fig — (TIF) [file pone.0198665.s002.tif]

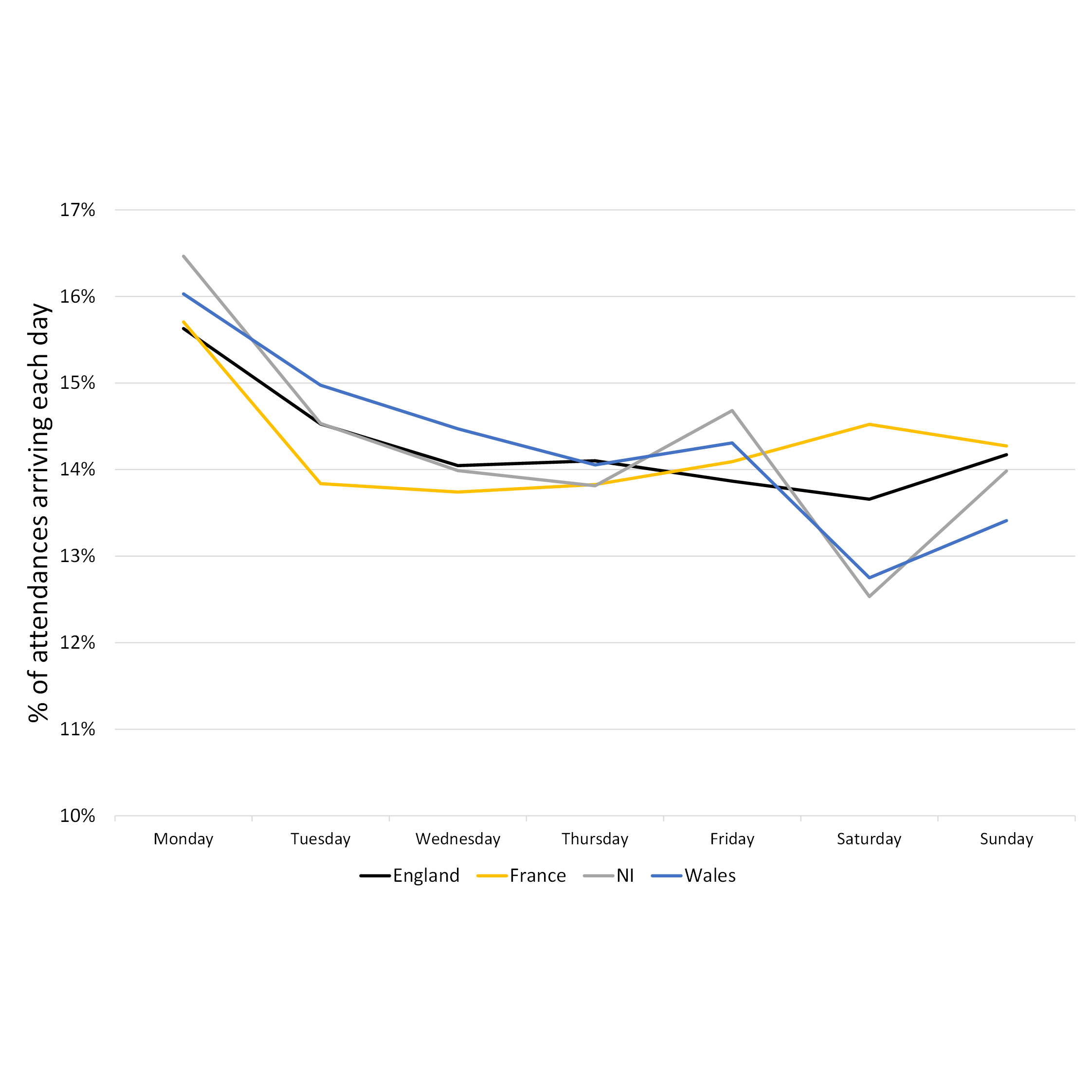

Supplement: S2 Fig — (TIF) [file pone.0198665.s003.tif]

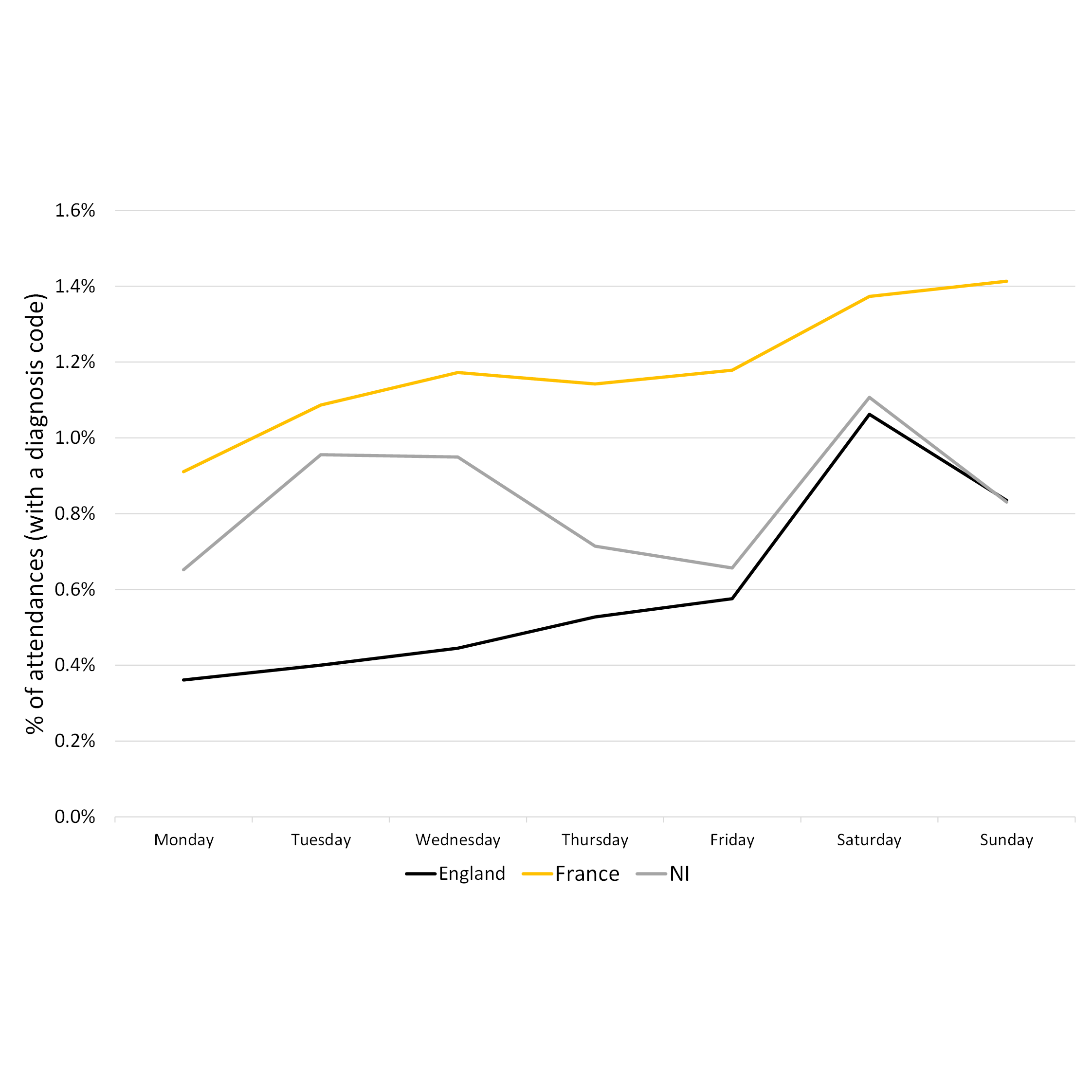

Supplement: S3 Fig — (TIF) [file pone.0198665.s004.tif]

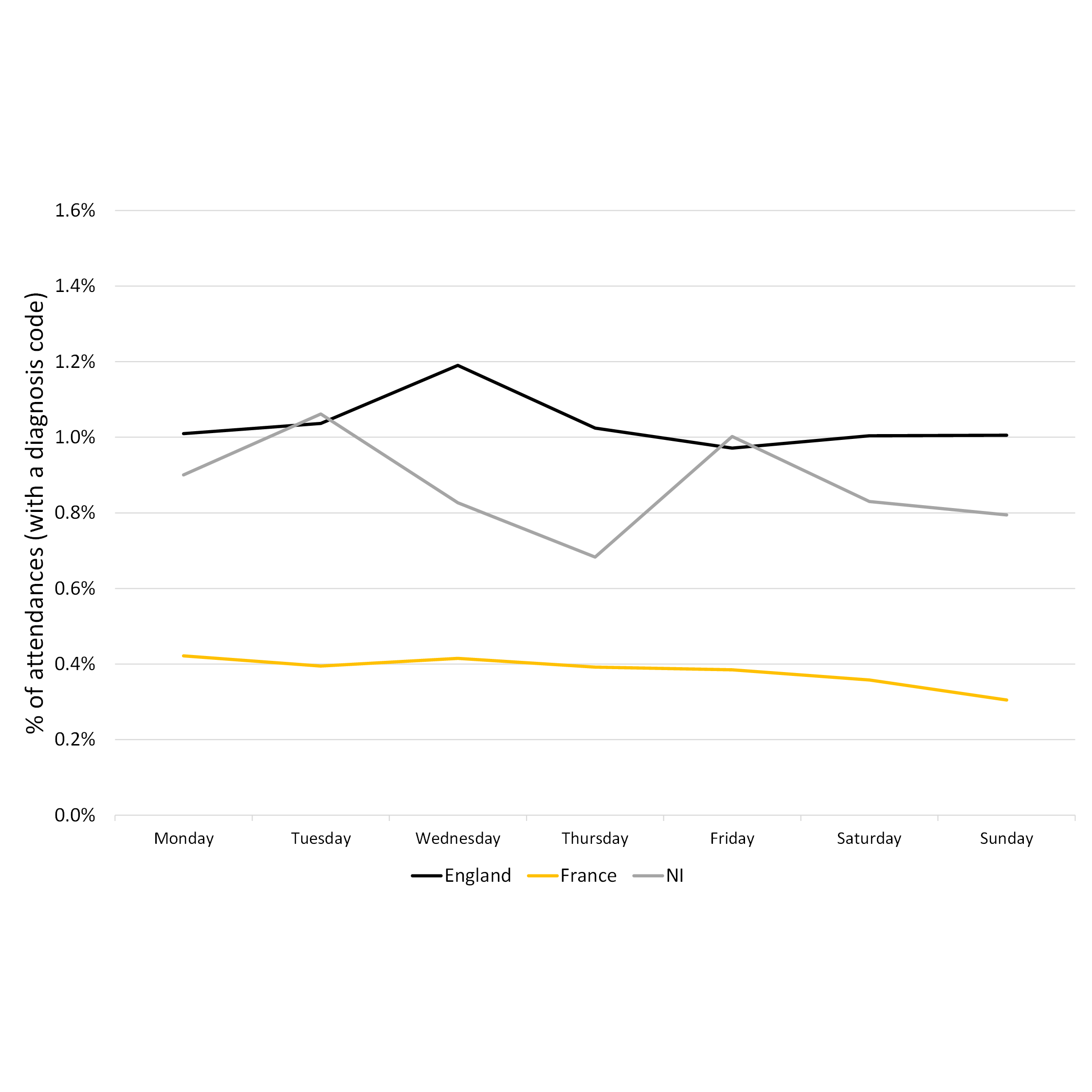

Supplement: S4 Fig — (TIF) [file pone.0198665.s005.tif]
